# Comparison of total prevalence, perinatal prevalence, and livebirth prevalence of birth defects in Hunan Province, China, 2016–2020

**DOI:** 10.3389/fpubh.2024.1297426

**Published:** 2024-09-11

**Authors:** Xu Zhou, Xiu Zeng, Junqun Fang, Jian He, Haiyan Kuang, Xinjun Hua, Aihua Wang

**Affiliations:** Hunan Provincial Maternal and Child Health Care Hospital, Changsha, Hunan, China

**Keywords:** congenital anomalies, epidemiology, total prevalence, livebirth prevalence, perinatal prevalence

## Abstract

**Objective:**

Birth defect of any type is undesirable and often pose a negative impact on the health and development of the newborn. Birth defects surveillance with datasets from surveillance health-related programs are useful to predict the pattern of birth defects and take preventive measures. In this study, the total prevalence, perinatal prevalence, and livebirth prevalence of birth defects were compared.

**Methods:**

Data were obtained from the Birth Defects Surveillance System in Hunan Province, China, 2016–2020. The total prevalence is the number of birth defects (including livebirths, stillbirths, and selective terminations of pregnancy) per 1,000 births (including livebirths and stillbirths). The perinatal prevalence is the number of birth defects (between 28 weeks gestation and 7 days postpartum) per 1,000 births. The livebirth prevalence is the number of liveborn birth defects per 1,000 births (unit: ‰). Underestimated proportion (unit: %) is the reduction level of perinatal prevalence or livebirth prevalence compared to the total prevalence. Prevalence with 95% confidence intervals (CI) was calculated using the log-binomial method. Chi-square tests (*χ*^2^) were used to examine if significant differences existed in prevalence or underestimated proportion between different groups.

**Results:**

A total of 847,755 births were included in this study, and 23,420 birth defects were identified, including 14,459 (61.74%) birth defects with gestational age > =28 weeks, and 11,465 (48.95%) birth defects in livebirths. The total prevalence, perinatal prevalence, and livebirth prevalence of birth defects were 27.63‰ (95%CI, 27.27–27.98), 17.06‰ (95%CI, 16.78–17.33), and 13.52‰ (95%CI, 13.28–13.77), respectively, and significant differences existed between them (*χ^2^* = 4798.55, *p* < 0.01). Compared to the total prevalence, the perinatal prevalence and livebirth prevalence were underestimated by 38.26 and 51.05%, respectively. Significant differences existed between the total prevalence, perinatal prevalence, and livebirth prevalence of birth defects in all subgroups according to year, sex, residence, and maternal age (*p* < 0.05). Significant differences existed between the total prevalence, perinatal prevalence, and livebirth prevalence for 17 specific defects: congenital heart defect, cleft lip-palate, Down syndrome, talipes equinovarus, hydrocephalus, limb reduction, cleft lip, omphalocele, anal atresia, anencephaly, spina bifida, diaphragmatic hernia, encephalocele, gastroschisis, esophageal atresia, bladder exstrophy, and conjoined twins (*p* < 0.05). In comparison, no significant difference existed between the total prevalence, perinatal prevalence, and livebirth prevalence for 6 specific defects: polydactyly, other external ear defects, syndactyly, hypospadias, cleft palate, and anotia/microtia (*p* > 0.05).

**Conclusion:**

The total prevalence and livebirth prevalence of birth defects in Hunan Province, China, was not well studied. A systematic study was conducted to compare the total prevalence, perinatal prevalence, and livebirth prevalence of birth defects. The study reveals that significant differences existed between the total prevalence, perinatal prevalence, and livebirth prevalence of birth defects (including many specific defects), and year, sex, residence, and maternal age had significant impacts on it. The outcomes of the study will help to take preventive measures for birth defects as well as benefit the people involving public health and policymakers to improve the current scenario.

## 1 Introduction

Birth defects are structural or functional anomalies at or before birth (1). The observed prevalence of birth defects is about 2–3% worldwide (2). The prevalence of birth defects is estimated at 4–6% in China (3). Because of the long life expectancy of patients with birth defects, birth defects have been a major problem in health care in terms of the resources required (2). Moreover, severe birth defects were associated with stillbirths or child deaths (4–6). WHO estimated that about 12.6% of neonatal deaths worldwide each year are related to birth defects (7). Therefore, research on birth defects is an important field of public health.

In China, most studies on the prevalence of birth defects are based on hospital-based surveillance, and the study population only includes fetuses and infants between 28 weeks gestation and 7 days postpartum (3). To our knowledge, there have been no national studies on birth defects in China recently. Some regions in China reported the prevalence of birth defects. E.g., the prevalence of birth defects was 188.94 per 10,000 births in in Hunan Province (2010–2020) (8), 13.55 per 1,000 births in Guilin, Guangxi Zhuang Autonomous Region (2018–2020) (9), 71.51 per 10,000 fetuses in Southern Jiangsu (2014–2018) (10), and 10.11‰ in Dalian, Liaoning Province (2006–2010) (11). Obviously, those prevalences were significantly lower than the estimated prevalence (4–6%) (3), and some were also lower than the observed prevalence worldwide (2). Partially, it may result from many birth defects diagnosed and terminated before 28 weeks of gestation (12). However, to our knowledge, there have been fewer studies on birth defects before 28 weeks of gestation in China (13).

In this study, there are three primary indicators to describe the prevalence of birth defects: total prevalence, livebirth prevalence, and perinatal prevalence. The total prevalence is the number of birth defects (including livebirths, stillbirths, and selective terminations of pregnancy) per 1,000 births (including livebirths and stillbirths). The livebirth prevalence is the number of liveborn birth defects per 1,000 births (14). The total prevalence may reflect the underlying environmental and genetic risk factors for birth defects, and the livebirth prevalence is particularly useful for health service purposes, as it measures birth defects needing health care. It has been widely used in previous studies. Since most studies in China report the prevalence of birth defects between 28 weeks gestation and 7 days postpartum, to make it easier to describe the prevalence, the perinatal prevalence is defined as number of birth defects (between 28 weeks gestation and 7 days postpartum) per 1,000 births.

In this study, the total prevalence, perinatal prevalence, and livebirth prevalence of birth defects were compared using data from the Birth Defects Surveillance System in Hunan Province, China, 2016–2020. This research will contribute to the field. First, as described earlier, most studies in China reported only the perinatal prevalence of birth defects, while the total prevalence and livebirth prevalence were rarely mentioned. Second, birth defects surveillance programs aim to ascertain birth defects among all pregnancy outcomes if possible (15). With the development in medical and economic conditions, it seems more appropriate to report the total prevalence rather than the perinatal prevalence. This study may contribute to surveillance program improvements. Third, as described earlier, it is of great public health significance to describe the epidemiology of total prevalence, perinatal prevalence, and livebirth prevalence of birth defects, which may contribute to public health policy improvements, risk factor studies, and etiological research on birth defects.

## 2 Methods

### 2.1 Data sources

In this study, data were obtained from the Birth Defects Surveillance System in Hunan Province, China, 2016–2020, which is run by the Hunan Provincial Health Commission and involves 52 representative registered hospitals in Hunan Province. Hunan Province is located in south-central China and has a resident population of about 65 million. These 52 hospitals are appropriately distributed throughout the province, with about 150,000 to 200,000 live births annually, accounting for 1/4 to 1/3 of the total live births.

The surveillance data were electronically collected, and doctors in the surveillance sites were responsible for collecting it. Surveillance data of all births (including livebirths and stillbirths) and birth defects (from the beginning of pregnancy to 7 days after birth) included demographic characteristics such as year, residence, sex, and maternal age.

Birth defects are classified into 23 specific defects and coded according to the International Classification of Diseases (Tenth Revision, ICD-10, codes Q00–Q99). The ICD codes for the 23 specific defects are as following: anencephaly (Q00), spina bifida (Q05), encephalocele (Q01), hydrocephalus (Q03), cleft palate (Q35), cleft lip (Q36), cleft lip-palate (Q37), anotia/microtia (Q17.2, Q16.0), other external ear defects (Q17), esophageal atresia (Q39), anal atresia (Q42), hypospadias (Q54), bladder exstrophy (Q64.1), talipes equinovarus (Q66.0), polydactyly (Q69), syndactyly (Q70), limb reduction (Q71, Q72), diaphragmatic hernia (Q79.0), omphalocele (Q79.2), gastroschisis (Q79.3), conjoined twins (Q89.4), Down syndrome (Q90), congenital heart defects (Q20–26) or ‘other’ (Q00–Q99, excluding the codes mentioned above).

### 2.2 Informed consents

The Health Commission of Hunan Province collects the surveillance data and has formulated the “Maternal and Child Health Monitoring Manual in Hunan Province.” In the “Maternal and Child Health Monitoring Manual in Hunan Province,” the government has emphasized the privacy policy for informed consent from the surveillance population. All works were performed following relevant guidelines and regulations. Informed consent was obtained from all surveillance populations and/or their legal guardian(s). Doctors obtain consent from pregnant women before collecting surveillance data, witnessed by their families and the heads of the obstetrics or neonatal departments. Doctors obtain consent from their parents or guardians for live births, witnessed by their families and the heads of the obstetrics or neonatal departments.

### 2.3 Ethics guideline statement

This study was approved by the Medical Ethics Committee of Hunan Provincial Maternal and Child Health Care Hospital (NO: 2023-S044). It is a retrospective study of medical records; all data were fully anonymized before access. Moreover, the patient records were de-identified before analysis. All works were performed following relevant guidelines and regulations.

### 2.4 Data quality control

To carry out surveillance, the Hunan Provincial Health Commission formulated the “Maternal and Child Health Monitoring Manual in Hunan Province.” Data were collected and reported by experienced doctors. To maintain data integrity and reduce error rates of surveillance data, the Hunan Provincial Health Commission asked the technical guidance departments to carry out comprehensive quality control each year. First, the surveillance hospital’s administration will carry out quality control for the surveillance data. Then, provincial, municipal, and county administrations will conduct regular quality control of the surveillance hospitals in their districts every year.

### 2.5 Definition of prevalence of birth defects

The total prevalence is number of total birth defects per 1,000 births (including livebirths and stillbirths); the perinatal prevalence is number of birth defects (between 28 weeks gestation and 7 days postpartum) per 1,000 births; the livebirth prevalence is number of liveborn birth defects per 1,000 births (unit: ‰). Underestimated proportion (unit: %) is the reduction level of perinatal prevalence or livebirth prevalence compared to the total prevalence.

### 2.6 Statistical analysis

The prevalence of birth defects with 95% confidence intervals (CI) was calculated using the log-binomial method. Chi-square tests (*χ^2^*) were used to examine if significant differences existed in prevalence or underestimated proportion between different groups. Chi-square trend tests (*χ^2^_trend_*) were used to determine trends in underestimated proportion by year.

Statistical analyses were performed using SPSS 18.0 (IBM Corp., NY, USA).

## 3 Results

### 3.1 Prevalence of birth defects in Hunan Province, China, 2016–2020

A total of 847,755 births were included in this study, and 23,420 birth defects were identified, including 14,459 (61.74%) birth defects with gestational age > =28 weeks, and 11,465 (48.95%) birth defects in livebirths.

The total prevalence, perinatal prevalence, and livebirth prevalence of birth defects were 27.63‰ (95%CI, 27.27–27.98), 17.06‰ (95%CI, 16.78–17.33), and 13.52‰ (95%CI, 13.28–13.77), respectively. Significant differences existed between the total prevalence, perinatal prevalence, and livebirth prevalence of birth defects (*χ^2^* = 4798.55, *p* < 0.01). Compared to the total prevalence, the perinatal prevalence and livebirth prevalence were underestimated by 38.26 and 51.05%, respectively.

The total prevalence, perinatal prevalence, and livebirth prevalence of birth defects by year, sex, residence, and maternal age are shown in Table 1. Significant differences existed between the total prevalence, perinatal prevalence, and livebirth prevalence of birth defects in all subgroups according to year, sex, residence, and maternal age (*p* < 0.05).

**Table 1:** Prevalence of birth defects in Hunan Province, China, 2016–2020.

Indicators	Total births	Total birth defects		Birth defects with gestational age > =28 weeks			Birth defects in livebirths			*χ^2^*	*p*
		*n*	Total prevalence (‰, 95%CI)	*n*	Perinatal prevalence (‰, 95%CI)	Underestimated (%)	*n*	Livebirth prevalence (‰, 95%CI)	Underestimated (%)		
Year											
2016	170,688	4,794	28.09 (27.29–28.88)	3,107	18.20 (17.56–18.84)	35.19	2,363	13.84 (13.29–14.40)	50.71	925.53	<0.01
2017	196,316	5,456	27.79 (27.05–28.53)	3,533	18.00 (17.40–18.59)	35.25	2,854	14.54 (14.00–15.07)	47.69	941.79	<0.01
2018	177,762	4,708	26.48 (25.73–27.24)	2,900	16.31 (15.72–16.91)	38.40	2,294	12.90 (12.38–13.43)	51.27	973.80	<0.01
2019	164,840	4,539	27.54 (26.73–28.34)	2,643	16.03 (15.42–16.65)	41.77	2,139	12.98 (12.43–13.53)	52.88	1050.68	<0.01
2020	138,149	3,923	28.40 (27.51–29.29)	2,276	16.47 (15.80–17.15)	41.98	1815	13.14 (12.53–13.74)	53.73	937.62	<0.01
Sex											
Male	448,288	13,291	29.65 (29.14–30.15)	8,783	19.59 (19.18–20.00)	33.92	7,164	15.98 (15.61–16.35)	46.10	2114.63	<0.01
Female	399,368	8,870	22.21 (21.75–22.67)	5,640	14.12 (13.75–14.49)	36.41	4,288	10.74 (10.42–11.06)	51.66	1797.30	<0.01
Unknown	99	1,259	–	36	–	–	13	–	–		
Residence											
Urban	342,178	11,454	33.47 (32.86–34.09)	7,208	21.07 (20.58–21.55)	37.07	6,129	17.91 (17.46–18.36)	46.49	1965.44	<0.01
Rural	505,577	11,966	23.67 (23.24–24.09)	7,251	14.34 (14.01–14.67)	39.40	5,336	10.55 (10.27–10.84)	55.41	2891.90	<0.01
Maternal age											
<20	13,711	409	29.83 (26.94–32.72)	272	19.84 (17.48–22.20)	33.50	184	13.42 (11.48–15.36)	55.01	91.09	<0.01
20–24	118,531	3,104	26.19 (25.27–27.11)	1846	15.57 (14.86–16.28)	40.53	1,333	11.25 (10.64–11.85)	57.06	807.22	<0.01
25–29	357,582	9,513	26.60 (26.07–27.14)	6,017	16.83 (16.40–17.25)	36.75	4,799	13.42 (13.04–13.80)	49.55	1801.43	<0.01
30–34	243,649	6,564	26.94 (26.29–27.59)	4,141	17.00 (16.48–17.51)	36.91	3,387	13.90 (13.43–14.37)	48.40	1196.27	<0.01
> = 35	114,282	3,830	33.51 (32.45–34.57)	2,183	19.10 (18.30–19.90)	43.00	1762	15.42 (14.70–16.14)	53.99	943.12	<0.01
Total	847,755	23,420	27.63 (27.27–27.98)	14,459	17.06 (16.78–17.33)	38.26	11,465	13.52 (13.28–13.77)	51.05	4798.55	<0.01

CI = confidence intervals.

Compared to the total prevalence, the perinatal prevalence was underestimated by 35.19, 35.25, 38.40, 41.77, and 41.98% from 2016 to 2020, respectively, showing an upward trend (*χ^2^_trend_* = 78.73, *p* < 0.01); the perinatal prevalence was underestimated by 33.92 and 36.41% for males and females, respectively, which was higher in females than in males (*χ^2^* = 14.60, *p* < 0.01); the perinatal prevalence was underestimated by 37.07 and 39.40% for urban areas and rural areas, respectively, which was higher in rural areas than urban areas (*χ^2^* = 13.49, *p* < 0.01); the perinatal prevalence was underestimated by 33.50, 40.53, 36.75, 36.91, and 43.00% for maternal age < 20, 20–24, 25–29, 30–34, and > =35, respectively, which was higher in maternal age 20–24 and > =35 than 25–29 (*χ^2^* = 61.38, p < 0.01). Compared to the total prevalence, the livebirth prevalence of birth defects had similar epidemiology as described above (Table 1).

### 3.2 Prevalence of specific defects

Significant differences existed between the total prevalence, perinatal prevalence, and livebirth prevalence for 17 specific defects: congenital heart defect, cleft lip-palate, Down syndrome, talipes equinovarus, hydrocephalus, limb reduction, cleft lip, omphalocele, anal atresia, anencephaly, spina bifida, diaphragmatic hernia, encephalocele, gastroschisis, esophageal atresia, bladder exstrophy, and conjoined twins (*p* < 0.05). In comparison, no significant difference existed between the total prevalence, perinatal prevalence, and livebirth prevalence for 6 specific defects: polydactyly, other external ear defects, syndactyly, hypospadias, cleft palate, and anotia/microtia (*p* > 0.05).

Compared to the total prevalence, the perinatal prevalence was underestimated by 100.00% for conjoined twins; the perinatal prevalence underestimated by more than 80% for 5 specific defects: cleft lip-palate (82.31%), Down syndrome (87.10%), anencephaly (92.92%), encephalocele (90.12%), and gastroschisis (83.11%); the perinatal prevalence underestimated by 50–80% for 6 specific defects: hydrocephalus (55.70%), limb reduction (51.47%), cleft lip (50.43%), omphalocele (76.49%), spina bifida (66.38%), and diaphragmatic hernia (58.25%); the perinatal prevalence underestimated by less than 50% for 5 specific defects: congenital heart defect (36.85%), talipes equinovarus (39.15%), anal atresia (9.02%), esophageal atresia (23.29%), and bladder exstrophy (33.33%).

Compared to the total prevalence, the livebirth prevalence was underestimated by 100.00% for conjoined twins; the livebirth prevalence was underestimated by more than 80% for 8 specific defects: cleft lip-palate (91.90%), Down syndrome (95.88%), hydrocephalus (93.06%), omphalocele (83.39%), anencephaly (98.75%), spina bifida (87.34%), encephalocele (98.26%), and gastroschisis (93.92%); the livebirth prevalence was underestimated by 50–80% for 6 specific defects: congenital heart defect (51.34%), limb reduction (73.36%), cleft lip (54.78%), diaphragmatic hernia (78.35%), esophageal atresia (53.42%), and bladder exstrophy (66.67%); the livebirth prevalence was underestimated by less than 50% for 2 specific defects: talipes equinovarus (48.75%), and anal atresia (21.72%) (Table 2).

**Table 2:** Prevalence of specific defects.

Specific defects	Total birth defects		Birth defects with gestational age > =28 weeks			Birth defects in livebirths			*χ^2^*	*p*
	*n*	Total prevalence (‰, 95%CI)	n	Perinatal prevalence (‰, 95%CI)	Underestimated (%)	*n*	Livebirth prevalence (‰, 95%CI)	Underestimated (%)		
Congenital heart defect	6,589	7.77 (7.58–7.96)	4,161	4.91 (4.76–5.06)	36.85	3,206	3.78 (3.65–3.91)	51.34	1315.03	<0.01
Polydactyly	1968	2.32 (2.22–2.42)	1888	2.23 (2.13–2.33)	4.07	1862	2.20 (2.10–2.30)	5.39	3.21	0.20
Cleft lip-palate	1,481	1.75 (1.66–1.84)	262	0.31 (0.27–0.35)	82.31	120	0.14 (0.12–0.17)	91.90	1804.03	<0.01
Other external ear defects	1,102	1.30 (1.22–1.38)	1,061	1.25 (1.18–1.33)	3.72	1,038	1.22 (1.15–1.30)	5.81	1.97	0.37
Down syndrome	1,093	1.29 (1.21–1.37)	141	0.17 (0.14–0.19)	87.10	45	0.05 (0.04–0.07)	95.88	1575.32	<0.01
Talipes equinovarus	802	0.95 (0.88–1.01)	488	0.58 (0.52–0.63)	39.15	411	0.48 (0.44–0.53)	48.75	151.43	<0.01
Syndactyly	662	0.78 (0.72–0.84)	626	0.74 (0.68–0.80)	5.44	605	0.71 (0.66–0.77)	8.61	2.64	0.27
Hypospadias	470	0.55 (0.50–0.60)	458	0.54 (0.49–0.59)	2.55	404	0.48 (0.43–0.52)	14.04	5.57	0.06
Hydrocephalus	447	0.53 (0.48–0.58)	198	0.23 (0.20–0.27)	55.70	31	0.04 (0.02–0.05)	93.06	389.08	<0.01
Limb reduction	443	0.52 (0.47–0.57)	215	0.25 (0.22–0.29)	51.47	118	0.14 (0.11–0.16)	73.36	215.30	<0.01
Cleft lip	345	0.41 (0.36–0.45)	171	0.20 (0.17–0.23)	50.43	156	0.18 (0.16–0.21)	54.78	98.57	<0.01
Omphalocele	319	0.38 (0.33–0.42)	75	0.09 (0.07–0.11)	76.49	53	0.06 (0.05–0.08)	83.39	292.62	<0.01
Cleft palate	278	0.33 (0.29–0.37)	254	0.30 (0.26–0.34)	8.63	247	0.29 (0.26–0.33)	11.15	2.04	0.36
Anal atresia	244	0.29 (0.25–0.32)	222	0.26 (0.23–0.30)	9.02	191	0.23 (0.19–0.26)	21.72	6.48	0.04
Anencephaly	240	0.28 (0.25–0.32)	17	0.02 (0.01–0.03)	92.92	3	0.00 (0.00–0.01)	98.75	408.10	<0.01
Spina bifida	229	0.27 (0.24–0.31)	77	0.09 (0.07–0.11)	66.38	29	0.03 (0.02–0.05)	87.34	195.27	<0.01
Anotia/microtia	209	0.25 (0.21–0.28)	185	0.22 (0.19–0.25)	11.48	179	0.21 (0.18–0.24)	14.35	2.64	0.27
Diaphragmatic hernia	194	0.23 (0.20–0.26)	81	0.10 (0.07–0.12)	58.25	42	0.05 (0.03–0.06)	78.35	117.98	<0.01
Encephalocele	172	0.20 (0.17–0.23)	17	0.02 (0.01–0.03)	90.12	3	0.00 (0.00–0.01)	98.26	274.93	<0.01
Gastroschisis	148	0.17 (0.15–0.20)	25	0.03 (0.02–0.04)	83.11	9	0.01 (0.00–0.02)	93.92	190.71	<0.01
Esophageal atresia	73	0.09 (0.07–0.11)	56	0.07 (0.05–0.08)	23.29	34	0.04 (0.03–0.05)	53.42	14.08	<0.01
Bladder exstrophy	30	0.04 (0.02–0.05)	20	0.02 (0.01–0.03)	33.33	10	0.01 (0.00–0.02)	66.67	10.00	0.01
Conjoined twins	23	0.03 (0.02–0.04)	0	0.00 (0.00–0.00)	100.00	0	0.00 (0.00–0.00)	100.00	46.00	<0.01

Total births: 847755.

CI = confidence intervals.

## 4 Discussion

Overall, significant differences existed between the total prevalence, perinatal prevalence, and livebirth prevalence of birth defects (including many specific defects), and year, sex, residence, and maternal age had significant impacts on it. There were several meaningful findings in this study.

First, compared to the total prevalence of birth defects (including many specific defects), the perinatal prevalence and livebirth prevalence were significantly underestimated. Li et al. found that the exclusion of pregnancy terminations <28 weeks of gestation resulted in a severe underestimation of the total prevalence of birth defects, particularly severe external defects (Shaanxi Province, China, 2014–2020) (13), which was consistent with findings in this study. As described in the introduction section, fewer studies on birth defects before 28 weeks of gestation in China, or compared the total prevalence, perinatal prevalence, and livebirth prevalence of birth defects. Some original contributions to the field were made in this study. E.g., Zhou et al. (8) found that the perinatal prevalence of birth defects showed a decreasing trend from 2010 to 2020. However, no significant trend in the total prevalence of birth defects was shown in this study. It indicates that an increasing number of birth defects were terminated before 28 weeks of gestation. The total prevalence of birth defects and most specific defects appeared consistent with the global prevalence (14, 16–33). However, the perinatal prevalence of birth defects and many specific defects were lower than the observed global prevalence. E.g., the total prevalence of Down syndrome was 1.29‰, consistent with the observed global prevalence (almost 1 in 600 live births) (26), while the perinatal prevalence of Down syndrome was only 0.17‰, almost tenfold lower than the observed global prevalence. It may result from well-established prenatal screening and diagnostic strategies for Down syndrome, and most Down syndrome are diagnosed in the first trimester or second trimester (34–36). It is indicated that the total prevalence, rather than the perinatal prevalence, should be used to reflect the underlying environmental and genetic risk factors for birth defects. Moreover, the prevalence of some specific defects was more likely to be underestimated, such as conjoined twins, anencephaly, encephalocele, down syndrome, gastroschisis, and cleft lip-palate, which were mainly major structural defects. The primary diagnostic method for birth defects in China is prenatal ultrasonography examination (37, 38). With the development of medical and economic conditions, more and more severe birth defects are diagnosed and terminated before 28 weeks gestation. The underestimated proportion of Down syndrome was also relatively higher, which may be mainly associated with prenatal screening and diagnosis of Down syndrome in the first trimester or second trimester (34–36). To increase the prenatal screening rate for Down syndrome, the Hunan Provincial Government has implemented a program to provide free Down syndrome screening in the second trimester for pregnant women since 2016, and almost all pregnant women in Hunan Province do it. In addition, the livebirth prevalence of birth defects (and a broad range of specific defects) was reported in this study, which has also been less addressed in previous studies in China. As mentioned in the Introduction section, the livebirth prevalence is particularly useful for health service purposes.

Second, compared to the total prevalence of birth defects, the underestimated proportions of perinatal prevalence and livebirth prevalence showed an upward trend from 2016 to 2020, which may be mainly related to medical and economic conditions. As explained above, with the development of medical and economic conditions, more and more severe birth defects are diagnosed and terminated before 28 weeks gestation.

Third, compared to the total prevalence of birth defects, the underestimated proportions of perinatal prevalence and livebirth prevalence were higher in females than in males. It is inconsistent with what is commonly believed. E.g., the major congenital heart defects (including transposition of great arteries, tetralogy of Fallot, and double outlet right ventricle), genital, urinary, musculoskeletal, and digestive disorders, and orofacial clefts were more common in males than females (39, 40). Theoretically, the proportion of deaths attributable to birth defects is higher in males than females if there is no significant difference in the prevalence of birth defects between males and females, which may lead to a higher underestimated proportion in males than females. Therefore, the underestimated proportion of prevalence was higher in females than in males, which may be mainly associated with the differences in total prevalence between males and females. In this study, the total prevalence of birth defects was significantly higher in males than females (29.65‰ vs. 22.21‰), which supported the conclusion in this study. In addition, the “boy preference” phenomenon exists in some areas of China, especially in poor rural areas (41), which may also partly contribute to more female than male fetuses with birth defects terminated.

Fourth, compared to the total prevalence of birth defects, the underestimated proportions of perinatal prevalence and livebirth prevalence were higher in rural areas than urban areas, which may also be mainly associated with medical and economic conditions. Urban areas are associated with better medical and economic conditions, and better medical and economic conditions are good for the survival of children with birth defects (42). Zhou et al. (43) found that perinatal deaths attributable to birth defects were more common in rural than urban areas. It also supported this conclusion.

Fifth, compared to the total prevalence of birth defects, the underestimated proportions of perinatal prevalence and livebirth prevalence were higher in maternal age 20–24 and > =35 than in 25–29. It may be mainly related to the fact that many severe defects are more common in fetuses with low and advanced maternal age (8, 44–47). In addition, low maternal age is associated with high reproductive ability and partly low economic conditions, encouraging mothers to terminate fetuses with birth defects and try to conceive healthy babies. It may partly contribute to the results.

Overall, the above outcomes will benefit public health and help policymakers improve the current scenario. E.g., as most studies in China reported only the perinatal prevalence of birth defects, findings in this study may prompt future studies to report the total prevalence and livebirth prevalence, and the policymakers may also be more likely to consider the total prevalence and livebirth prevalence when developing public health policies. The above outcomes on the epidemiology of birth defects may be helpful for future studies on risk factors and etiology of birth defects, and policymakers may be more likely to implement interventions for high-risk populations.

Some things could be improved in this study. First, although significant differences between the total prevalence, perinatal prevalence, and livebirth prevalence of birth defects were found, the mechanism was unclear due to data limitations. Further research is needed. Second, birth defects beyond 7 days after birth were not included in this study. Third, some potential factors associated with the underestimated proportions of prevalence were not included due to data limitations, such as paternal age and educational background. Fourth, many fetuses had multiple specific defects, which may be associated with the underestimated proportions of prevalence. However, it was not analyzed in this study.

## 5 Conclusion

The total prevalence and livebirth prevalence of birth defects in Hunan Province, China, was not well studied. A systematic study was conducted to compare the total prevalence, perinatal prevalence, and livebirth prevalence of birth defects. The study reveals that significant differences existed between the total prevalence, perinatal prevalence, and livebirth prevalence of birth defects (including many specific defects), and year, sex, residence, and maternal age had significant impacts on it. The outcomes of the study will help to take preventive measures for birth defects as well as benefit the people involving public health and policymakers to improve the current scenario.

## Data availability statement

The original contributions presented in the study are included in the article/supplementary material, further inquiries can be directed to the corresponding authors.

## Ethics statement

The studies involving humans were approved by the Medical Ethics Committee of Hunan Provincial Maternal and Child Health Care Hospital. The studies were conducted in accordance with the local legislation and institutional requirements. The ethics committee/institutional review board waived the requirement of written informed consent for participation from the participants or the participants’ legal guardians/next of kin because Since the Health Commission of Hunan Province collects those data, and the government has emphasized the privacy policy in the “Maternal and Child Health Monitoring Manual in Hunan Province,” there is no additional written informed consent.

## Author contributions

XZh: Conceptualization, Data curation, Formal analysis, Methodology, Project administration, Supervision, Validation, Visualization, Writing – original draft, Writing – review & editing. XZe: Writing – review & editing, Methodology, Supervision, Project administration, Resources. JF: Data curation, Writing – review & editing. JH: Data curation, Writing – review & editing. HK: Data curation, Writing – review & editing. XH: Data curation, Writing – review & editing. AW: Data curation, Writing – review & editing.
